# Diagnostic accuracy of post-mortem cardiovascular magnetic resonance imaging in fetuses, newborns, and children

**DOI:** 10.1186/1532-429X-14-S1-O54

**Published:** 2012-02-01

**Authors:** Sudhin Thayyil, Neil Sebire, Michael A Ashworth, Andrew M Taylor

**Affiliations:** 1 Centre for Cardiovascular Imaging, UCL Institute of Cardiovascular Science, London, United Kingdom; 2 Academic Neonatology, UCL Institute for Women’s Health, London, United Kingdom; 3 Histopathology, UCL Institute of Child Health, London, United Kingdom; 4 Histopathology, Great Ormond Street Hospital for Children, London, United Kingdom

## Background

Whole body magnetic resonance (MR) imaging is increasingly used as an alternative for conventional autopsy; however, a recent systematic review suggested that post-mortem MR imaging had a sensitivity of 12% (95% CI-0.4 to 31) for detecting major cardiac pathology (Thayyil et al. Eur J Radiol 2010; 75(1):e142-8). We wanted to compare the accuracy of high-resolution 3D post-mortem cardiovascular MR (CMR) imaging with conventional cardiac autopsy in fetuses, newborns and children.

## Methods

We prospectively studied 342 fetuses, newborns and children, referred for autopsy to Great Ormond Street Hospital for Children or University College London Hospitals, over a 3-year period. We acquired high resolution 3D post-mortem CMR images using T2-weighted turbo spin echo and 3D constructive interference in steady state (CISS) sequences at 1.5 T MR (Avanto, Siemens Medical Systems, Erlangen, Germany), before autopsy. A specialist paediatric CMR imager reported the CMR images blinded to the autopsy data. Experienced paediatric pathologists performed the conventional autopsy according to the Royal College of Pathologist UK guidelines, blinded to the CMR data. All data were entered into a database using predefined coded categorical variables. The study was approved by GOSH/ICH research ethics committee.

## Results

**Figure 1:**
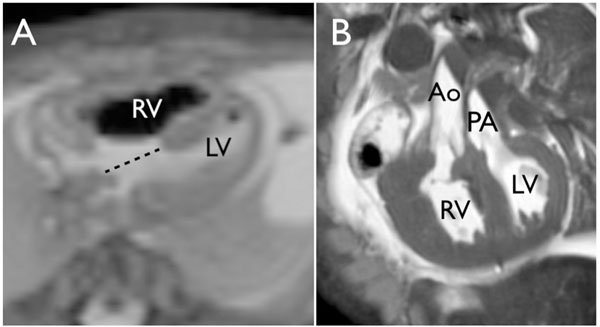
A - 4-chamber view showing a complete atrioventricular septal defect (dotted line) in an 18-week fetus; B - Oblique sagittal view showing transposition of the great arteries in a neonate.

Post-mortem CMR imaging was non-diagnostic in 34 (10%) of the cases (all fetuses). Of these 2 fetuses had congenital heart disease at autopsy; the remaining were normal. The data were subsequently analysed excluding the non-diagnostic cases. The sensitivity, specificity, positive predictive value (PPV) and negative predictive value (NPV) of CMR compared to conventional autopsy are shown in Table 1, with examples of image quality shown in Figures 1 & 2. Eight cases of significant heart disease were missed by MR imaging; of these five were in fetuses < 24 weeks (2 major and 3 minor congenital abnormalities) and 3 in children (all 3 myocarditis).

**Table 1:** Diagnostic accuracy of post-mortem CMR.

Age group	Sensitivity %(95% CI)	Specificity %(95% CI)	PPV % (95% CI)	NPV % (95% CI)
<=24 Weeks	73 (45, 91)	94 (88, 98)	61 (39, 80)	97 (92, 99)
>24 weeks and newborns	95 (67, 100)	96 (90, 98)	67 (42, 89)	100 (96, 100)
Infants and Children	63 (31, 86)	97 (85, 100)	83 (44, 97)	92 (78, 97)

## Conclusions

High resolution 3D post-mortem CMR imaging, reported by a specialist paediatric CMR imager can accurately detect structural heart diseases in fetuses > 24 weeks, neonates and children, and the majority of abnormalities in those < 24 weeks. In older children, structural abnormalities were easily identified, but 3 cases of myocarditis were missed. Development of further CMR imaging methods is required to rule out myocarditis.

## Funding

UK Department of Health, UK National Institute of Health Research (NIHR), British Heart Foundation (BHF).

